# Evaluation of the Use of a Novel Intelligent Diagnosis and Cost Control System on Pediatric Bronchopneumonia Outcomes: Retrospective Cohort Study

**DOI:** 10.2196/74964

**Published:** 2025-10-15

**Authors:** Yanjun Wu, Kaijie Liu, Xinli Mao, Danjie Wu, Feng Zhu

**Affiliations:** 1 Department of Medical Affairs, Taizhou Hospital of Zhejiang Province, 150 Ximen Street, Linhai City, 317000, China, 86 0576-85120120; 2 Department of Pediatrics, Taizhou Hospital of Zhejiang Province, Linhai City, Zhejiang Province, China; 3 Department of Gastroenterology, Taizhou Hospital of Zhejiang Province, Linhai City, Zhejiang Province, China; 4 Department of Stomatology, Taizhou Hospital of Zhejiang Province, Linhai City, Zhejiang Province, China

**Keywords:** intelligent diagnosis and cost control system, clinical decision support system, clinical pathway, diagnosis-related group, pediatric bronchopneumonia, evaluation, medical expenses, treatment outcomes

## Abstract

**Background:**

Health care systems face challenges of inconsistent quality, inefficiency, and rising costs. Fragmented applications of clinical decision support systems (CDSSs), clinical pathways (CPs), and diagnosis-related group (DRG) payment systems have limited their synergistic potential.

**Objective:**

This study proposed a CDSS-CP-DRG closed-loop model enabled by digital health technologies; specifically, the CDSS optimized CP execution through real-time data, the CP standardized workflows to support DRG cost control, and DRG payment pressures drove iterative improvements in both technology and processes. This research aimed to validate the model’s effectiveness in clinical efficacy, cost control, and standardized diagnosis and treatment of bronchopneumonia in children and provide evidence for value-based health care transformation.

**Methods:**

A total of 4543 children with bronchopneumonia were selected and divided into the experimental or control group based on whether the intelligent diagnosis and cost control system was used in the diagnostic process. Chi-square test, 1-way analysis of variance, paired *t* test, multiple regression analysis, and other mathematical statistical methods were used to verify the difference between the outcomes of the two groups of patients.

**Results:**

This study demonstrated comparably high cure rates in both groups (*P*>.05). However, the experimental group exhibited a 0.4-day reduction in average length of stay, 12.3% lower total hospitalization costs, RMB 135.3 (US $19) higher medical insurance reimbursement surplus, and a reduction of 0.16 defined daily doses of antibiotic use intensity versus the control group (*P*<.05 for all significant differences).

**Conclusions:**

The novel intelligent diagnosis and cost control system demonstrated significant improvement in clinical effect, cost control, and standardized treatment for pediatric bronchopneumonia, but the CP for pediatric pneumonia requiring intensive care still needs further attention and adjustment.

## Introduction

### Background

Bronchopneumonia, a prevalent lower respiratory tract infection, exhibits high incidence in children globally [1], with approximately 150 million annual cases among those aged 0‐5 years. Severe cases requiring hospitalization account for 12.82% of affected children [2,3], imposing substantial health burdens and socioeconomic costs [4].

Clinical decision support systems (CDSSs) integrate patient-specific data with computerized knowledge bases to generate evidence-based recommendations through software algorithms, representing a pivotal advancement in health informatics [5,6]. Pediatric applications demonstrate the capacity of CDSSs to enhance diagnostic accuracy, reduce errors, and improve workflow efficiency via intelligent alerts [7]. In addition, standardization of treatment through the use of clinical pathways (CPs) has proven to be an effective way to streamline care in a way that minimizes errors and cost while improving outcomes [8].

Although these information-based systems hold transformative potential for global health care digitalization, rigorous economic evaluations and real-world effectiveness analyses remain scarce, with most evidence derived from narrative reviews rather than empirical data.

### The Design and Implementation Path of a Novel Intelligent Diagnosis and Cost Control System

**Figure 1.**
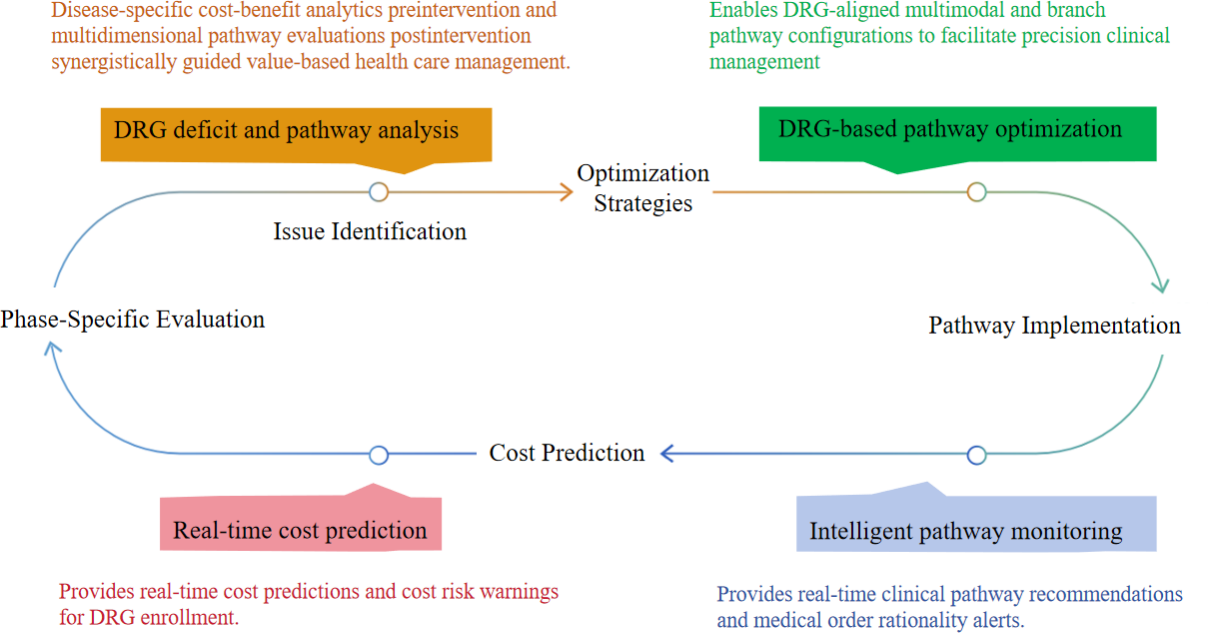
Closed-loop management framework of the intelligent decision and cost control system. DRG: diagnosis-related group.

As shown in Figure 1, the artificial intelligence (AI)–driven CP optimization and cost containment system uses generalizable identification mechanisms and data-intelligent decision-making, integrating natural language processing, data mining, and machine learning technologies. It is integrated with core hospital information systems including electronic medical records, the laboratory information system, and the picture archiving and communication system. The system constructs disease-specific smart CPs that synchronize treatment quality assurance with expenditure control. This machine learning–powered architecture enables granular diagnosis-related group (DRG) adaptation, achieving precision treatment protocols (dynamic pathway optimization based on real-time patient status monitoring), intelligent cost governance (automated detection of billing deviations through natural language processing–based charge item analysis), and quality-cost balance (continuous learning from multisource clinical data to maintain care standards while reducing low-value services).

### Implementation Pathway and Core Functions

The intelligent diagnosis and cost control system (IDCCS) operates through a streamlined workflow, integrating multisource data from hospital systems (eg, hospital information system, laboratory information system, picture archiving and communication system, electronic medical records), followed by automated data cleaning and standardization. It features customizable CPs with DRG-based treatment branches and preimplementation cost simulations using historical data. The system establishes baselines through retrospective data analysis, then automatically enrolls eligible patients into pathways. During treatment, it provides real-time clinical decision support with deviation alerts requiring clinician confirmation, while monitoring prescription appropriateness and forecasting costs with early warnings. Posttreatment multidimensional analytics evaluate pathway efficacy, supporting continuous optimization.

### Study Goal

This retrospective study analyzed the clinical outcomes, cost containment, and treatment standardization metrics of children with bronchopneumonia in a tertiary hospital in Eastern China (May 2023 to November 2024) and compared the difference of using or not using the IDCCS on these treatment outcome indicators. The findings provided empirical evidence for developing specialized and systematic intelligent CDSSs integrating cost-effective quality control in pediatric respiratory treatment.

## Methods

### Study Design

We designed a retrospective cohort study to analyze the differences in the results of 3 dimensions of treatment outcomes of hospitalized children with pneumonia for whom the IDCCS was used or was not used. The specific indicators of the 3 dimensions were as follows. The indicator of clinical efficacy was whether the disease improved. The economic indicators included length of hospital stay, total hospitalization expenses, proportion of drugs and consumables in expenses, and medical insurance settlement balance. The standardized diagnosis and treatment index was the volume of antibiotics dispensed (defined daily dose [DDD]).

Antibiotic therapy remains pivotal in pediatric pneumonia management [9]. The selection of DDD for evaluating antibiotic therapy compliance and normative treatment aligns with international guidelines [10,11]. This metric demonstrates superior validity in quantifying adherence to evidence-based protocols while balancing clinical relevance, operational feasibility, and comparability across multicenter studies [12]. The quantitative indicators including length of stay and health care costs can serve as economic end points to further validate the IDCCS’s beneficial effects on treatment outcomes through cost analysis and resource utilization evaluation. These metrics’ integration with CDSSs enables real-time feedback.

### IDCCS Evaluation

#### Participants and Setting

This study was carried out at a tertiary hospital in Eastern China. The deployment of the IDCCS in December 2023 served as a natural intervention. Accordingly, patients retrospectively enrolled from the preimplementation phase (January to November 2023) comprised the control group. Conversely, patients from the postimplementation phase (the corresponding period in 2024) comprised the experimental group.

Information was collected for patients admitted to the hospital with the diagnosis of “bronchial pneumonia” and aged <14 years. The experimental group consisted of patients for whom the IDCCS was used (n=1658), and the control group consisted of patients for whom the IDCCS was not used (n=2885).

Cases were excluded if their medical records had home pages with incomplete information or important fields missing, home pages with errors, hospitalization expenses with outliers, hospitalization expenses below 100 yuan (US $13.95), hospitalization days below 0.1 days; ultimately, records for 4543 children were retained.

#### Data Collection

All the data were extracted from the hospital’s internal database. To describe the population included in the study, the following variables were extracted at baseline: age, sex, diagnosis, with coinfection or not, and treatment result.

### Statistical Analysis

Statistical analyses were conducted using SPSS for Windows (version 23.0; IBM Corp). Categorical data were denoted in numbers and percentages, while continuous variables were represented as means and SDs. Data normality and homoscedasticity were preliminarily verified before executing each statistical analysis.

The *χ*^2^ test was used to compare the differences in categorical variable indicators between the experimental group and the control group (gender, diagnosis results of disease severity, concurrent infections, treatment outcomes). Paired *t* test and 1-way ANOVA were used to test the differences in continuous variables of the research subjects (length of hospital stay, total hospitalization cost, proportion of drug and consumable costs in the total cost, balance of medical insurance settlement, DDD).

For all tests, a *P* value ≤.05 was considered statistically significant.

Furthermore, in the comparison of treatment outcome differences, we not only conducted the analysis of the overall experimental group and intervention group, but also further divided the patients based on whether they required intensive care and whether they had severe complications or comorbidities, and then conducted further analysis separately.

### Ethical Considerations

This study, conducted in accordance with the Declaration of Helsinki, received approval from the institutional review board of Taizhou Hospital of Zhejiang Province (K20250873). As it was a retrospective study involving the analysis of existing, anonymized data, the requirement for informed consent was waived by the ethics committee. To ensure data security and patient confidentiality, all personally identifiable information was removed prior to analysis. The data were accessed strictly for research purposes and stored on secure, password-protected hospital servers [13].

## Results

### Overview

Among the 4543 patients included in the study, 1658 were in the IDCCS-used (experimental) group, and 2885 were in the IDCCS-without (control) group. Data on demographic characteristics, discharge diagnosis, coinfection with or without, and treatment outcomes of the patients are provided in Table 1. No statistically significant differences were observed in age or sex distribution between the experimental and control groups (*P>*.05).

**Table 1.** Characteristics of participants in the study sample (N=4543).

Characteristics		Control (n=2885)	Experimental (n=1658)	Total missing, n (%)	*F* test/chi-square (*df*)
	Age (years), mean (SD)	4.17 (3.16)	3.54 (3.12)	0 (0)	1.9 (1, 4543)^a^
	Sex (female), n (%)	772 (46.6)	596 (48)	1643 (36.2)	0.4 (1, 1368)^b^
Discharge diagnosis					
	Pediatric pneumonia	2577 (89.3)	1648 (99.4)	0 (0)	165.0^c^ (2, 4413)^b^
	Pediatric pneumonia requiring intensive care	178 (6.2)	10 (0.6)		
Coinfection					
	Without	2324 (80.6)	1067 (64.4)	0 (0)	146.0^c^ (2, 4543)^b^
	With	561 (19.4)	591 (35.6)		
Treatment outcome					
	Improved	2840 (99.27)	1647 (99.34)	24 (0.5)	2.5 (2, 4159)^b^
	Unresolved	22 (0.77)	9 (0.54)		
	Death	0 (0)	1 (0.06)		

a *F* test.

b Chi-square test.

c *P*<.001.

### Clinical Efficacy

Participants were stratified into 3 diagnostic subgroups: pediatric pneumonia, pediatric pneumonia requiring intensive care, and pediatric pneumonia with severe comorbidities and complications. Statistical analysis revealed no significant differences in treatment outcomes between the intervention and control groups across these subgroups (*P*>.05) (Table 2).

**Table 2.** The differences in treatment outcomes among participants in different diagnostic subgroups.

		Control (n=2554)	Experimental (n=1647)	Chi-square (*df*)
Pediatric pneumonia				
	Improved	2534 (99.2)	1637 (99.4)	2.4 (2)
	Unresolved	20 (69)	9 (31)	
	Death	0 (0)	1 (0.1)	
Pediatric pneumonia requiring intensive care				
	Improved	176 (98.9)	10 (100)	0.1 (1)
	Unresolved	2 (1.1)	0 (0)	
Pediatric pneumonia with severe comorbidities and complications				
	Improved	130 (100)	—^a^	

a Not applicable.

### Economic Indicators

As shown in the analytical data in Table 3, following the implementation of the IDCCS, the experimental groups demonstrated a statistically significant reduction in the length of stay by 0.4 days (*P*<.001), with total hospitalization expenses decreasing by 12.2% (RMB 508.56 [US $71.43]) and medical insurance settlement balance increasing by RMB 135.3 (US $19; both *P*<.001).

Further group analysis found that the previously mentioned indicators of patients with pneumonia in the experimental group were improved. However, no significant differences were observed in the pediatric pneumonia subgroup requiring intensive care (*P*>.05).

**Table 3.** Differences in economic indicators of all participants.

		Control (n=2885)			Experimental (n=1658)				
		Mean	SD	95% CI	Mean	SD	95% CI	*F* test (*df*)	*P* value
Length of hospital stay		6.5	0.0	6.4 to 6.6	6.1	0.0	6.0 to 6.2	32.7 (1)	<.001
	Pediatric pneumonia	6.3	0.0	6.2 to 6.4	6.1	0.0	6.0 to 6.2	10.2 (1)	.001
	Pediatric pneumonia requiring intensive care	7.9	0.0	7.5 to 8.3	8.9	0.0	7.7 to 10.4	1.4 (1)	.24
Total hospitalization expenses		4153.9	0.2	4086.2 to 4225.9	3645.4	1.1	3588.8 to 3711.6	104.5 (1)	<.001
	Pediatric pneumonia	3824.4	–0.3	3770.5 to 3884.0	3610.4	–0.7	3559.0 to 3663.5	28.9 (1)	<.001
	Pediatric pneumonia requiring intensive care	7266.5	–3.0	6829.9 to 7748.0	9410.2	20.89	7578.5 to 11,828.3	4.5 (1)	.04
Proportion of drugs and consumables in expenses		0.1	0.0	0.1 to 0.1	0.1	0.0	0.1 to 0.1	0.5 (1)	.49
	Pediatric pneumonia	0.1	0.0	0.1 to 0.1	0.1	0.0	0.1 to 0.1	4.0 (1)	.046
	Pediatric pneumonia requiring intensive care	0.1	0.0	0.1 to 0.2	0.1	0.0	0.1 to 0.2	1.3 (1)	.25
Medical insurance settlement balance		–457.0	–0.4	–508.6 to –405.1	−321.7	−1.7	–377.0 to –272.0	11.8 (1)	<.001
	Pediatric pneumonia	–533.7	–0.7	–581.7 to –485.4	193.6	1.0	–390.8 to –290.1	28.3 (1)	<.001
	Pediatric pneumonia requiring intensive care	715.0	–5.0	381.6 to 1052.1	2704.7	12.4	135.8 to 5335.7	6.1 (1)	.01

### Standardized Diagnosis and Treatment

Table 4 demonstrates that while the experimental group showed a modest reduction in mean antibiotic prescribing intensity (−0.1 DDD) compared to the control group, this system-wide effect was not statistically significant (*P*=.21). However, in the pediatric pneumonia subgroup that leveraged the clinical decision support features of the IDCCS, we observed a statistically and clinically meaningful reduction in antimicrobial exposure intensity (−0.2 DDD; *P*=.002), representing a 6.8% decrease compared to the control group.

**Table 4.** Differences in antibiotic use intensity among participants.

		Control (n=2588)			Experimental (n=1613)				
		Mean	SD	95% CI	Mean	SD	95% CI	*F* test (*df*)	*P* value
All participants		2.3	0.0002	2.3 to 2.4	2.3	−0.0006	2.2 to 2.4	1.6 (1)	.21
Discharge diagnosis		2.3	−0.0004	2.2 to 2.3	2.3	−0.0010	2.2 to 2.3	0.0 (1)	.97
	Pediatric pneumonia	2.3	0.0020	2.1 to 2.6	2.2	−0.0132	2.0 to 2.3	9.8 (1)	.002
	Pediatric pneumonia requiring intensive care	2.4	0.0010	2.3 to 2.5	2.3	0.0004	2.2 to 2.4	1.7 (1)	.19
Coinfection		2.1	0.0029	1.9 to 2.3	2.2	−0.0021	2.1 to 2.3	1.1 (1)	.30
	Without	2.3	0.0002	2.3 to 2.4	2.3	−0.0006	2.2 to 2.4	1.6 (1)	.21
	With	2.3	−0.0004	2.2 to 2.3	2.3	−0.0010	2.2 to 2.3	0.0 (1)	.97

## Discussion

### Principal Results

Our findings demonstrate that implementing the IDCCS CDSS significantly reduced treatment costs and improved standardized treatment in pediatric pneumonia management [14]. The digital intervention was associated with a reduction in the length of stay, a decrease in total hospitalization expenses, an increase in the medical insurance settlement balance, and a reduction in antibiotic prescribing volumes [15].

These results highlight the dual benefits of AI-driven decision support in enhancing value-based treatment while maintaining normative treatment quality.

However, within our study, in the pediatric pneumonia subgroup requiring intensive care, the aforementioned outcome metrics demonstrated no statistically significant improvement, suggesting that the current CP algorithms in the intelligent diagnosis assistance system require further optimization for use in critically ill patient populations.

### Interpretation and Comparison With Prior Work

Extensive international research demonstrates that health information technology, characterized by accessibility, feasibility, and efficiency, significantly reduces antibiotic resistance rates, antimicrobial therapy mortality, and health care costs [16-19].

Christensen et al [20] put forward that CDSSs were designed according to the “Five Rights” principle: delivering the right information to the right recipient at the right time, through the right channel, in the right format. As a tool for auxiliary diagnosis, CDSS can improve the quality and efficiency of diagnosis. Düvel et al [21] demonstrated in a qualitative study that CDSS holds promise for optimizing antibiotic stewardship, while current systems lack AI technology integration.

Umoh et al [22] proposed the future development of CDSS in clinical treatment and management; a comprehensive tool that is precise and user-friendly would improve clinical decisions and efficiency.

Farkas et al [23] revealed that the use of CPs could reduce unnecessary diagnostic testing in hospitalized patients, which is consistent with the conclusion of our study. Jaafar et al [24] found that the implementation of DRG policy and CPs significantly enhanced health care resource utilization. However, evidence evaluating the effectiveness of CPs using real-world data remains limited in current literature. As noted by Bakel et al [25], most hospitals were unable to measure their pathway outcomes or demonstrate improvement in care.

In these prior studies, CDSS, CP, and DRG payment systems were mostly applied and evaluated separately, and the fragmented application greatly limited their synergy potential. Current literature lacks real-world data studies investigating the integration of CDSSs with CPs.

This study innovatively proposed a CDSS-CP-DRG closed-loop model enabled by digital health technologies, where CDSS optimized CP execution through real-time data, CPs standardized workflows to support DRG cost control, and DRG payment pressures drove iterative improvements in both technology and processes. Their integration promoted evidence-based practice, reduced clinical variation, and curbed overutilization, particularly in resource-sensitive pediatric settings.

### Strengths and Limitations

DRG is a well-known type of hospital payment system that aims to establish the reference interval of medical expenses. It can effectively reduce costs, decrease the difficulty of standardized management of the medical diagnosis and treatment, and facilitate the macroprediction and control of medical expenses.

The ultimate goal of the DRG cost pathway is to achieve optimal medical value [26]. In this study, we integrated CDSS with CP systems using AI technology to develop the IDCCS, and the framework established a closed-loop quality and cost control process spanning preimplementation DRG-based deficit analysis and CP optimization, intraimplementation execution with real-time alerts, and postimplementation statistical analytics. This system ensures standardized diagnosis and treatment while managing and controlling health care costs for hospitalized patients through the establishment of defined insurance payment quotas. As a result, hospitals are incentivized to reduce medical costs proactively, shorten hospital stays, and minimize unnecessary expenditures in pursuit of economic profitability. This approach facilitates cost control and promotes the rational and standardized use of medical resources [27]. Ultimately, it aims to achieve low-cost, high-quality, efficient, and health-oriented value-based treatment [28,29].

### Conclusion

This study innovatively integrated a CDSS with CPs to optimize DRG-based case management. An AI-driven CDSS-CP framework was developed for pediatric pneumonia, demonstrating scalability for diverse clinical contexts. AI-optimized CPs enhanced cost-effectiveness (ie, reduced medical expenditures) and care standardization, further validating its role in health care system reform. The model contributed to evidence-based global health care practices and population health improvement. However, CPs for critical cases or other diseases require continuous optimization and adaptation to enhance health care resource utilization and standardize clinical practices.

## Abbreviations

AI: artificial intelligence
CDSS: clinical decision support system
CP: clinical pathway
DDD: defined daily dose
DRG: diagnosis-related group
IDCCS: intelligent decision and cost control system
